# A decision support framework for the discrimination of children with controlled epilepsy based on EEG analysis

**DOI:** 10.1186/1743-0003-7-24

**Published:** 2010-06-02

**Authors:** Vangelis Sakkalis, Tracey Cassar, Michalis Zervakis, Ciprian D Giurcaneanu, Cristin Bigan, Sifis Micheloyannis, Kenneth P Camilleri, Simon G Fabri, Eleni Karakonstantaki, Kostas Michalopoulos

**Affiliations:** 1 Biomedical Informatics Lab, Institute of Computer Science, Foundation for Research and Technology, Heraklion, Greece; 2 iBERG, Department of Systems and Control Engineering, Faculty of Engineering, University of Malta, Msida, Malta; 3 Department of Electronic and Computer Engineering, Technical University of Crete Chania, Greece; 4 Department of Signal Processing, Tampere University of Technology, Tampere, Finland; 5 Ecological University of Bucharest, Romania; 6 Clinical Neurophysiology Laboratory (L. Widen), Faculty of Medicine, University of Crete, Heraklion, Greece

## Abstract

**Background:**

In this work we consider hidden signs (biomarkers) in ongoing EEG activity expressing epileptic tendency, for otherwise normal brain operation. More specifically, this study considers children with controlled epilepsy where only a few seizures without complications were noted before starting medication and who showed no clinical or electrophysiological signs of brain dysfunction. We compare EEG recordings from controlled epileptic children with age-matched control children under two different operations, an eyes closed rest condition and a mathematical task. The aim of this study is to develop reliable techniques for the extraction of biomarkers from EEG that indicate the presence of minor neurophysiological signs in cases where no clinical or significant EEG abnormalities are observed.

**Methods:**

We compare two different approaches for localizing activity differences and retrieving relevant information for classifying the two groups. The first approach focuses on power spectrum analysis whereas the second approach analyzes the functional coupling of cortical assemblies using linear synchronization techniques.

**Results:**

Differences could be detected during the control (rest) task, but not on the more demanding mathematical task. The spectral markers provide better diagnostic ability than their synchronization counterparts, even though a combination (or fusion) of both is needed for efficient classification of subjects.

**Conclusions:**

Based on these differences, the study proposes concrete biomarkers that can be used in a decision support system for clinical validation. Fusion of selected biomarkers in the Theta and Alpha bands resulted in an increase of the classification score up to 80% during the rest condition. No significant discrimination was achieved during the performance of a mathematical subtraction task.

## Background

Epilepsy is one of the most common neurological disorders in childhood [1]. There are many epidemiological studies referring to the incidence of seizures. The average annual rate of new cases per year (incidence) of epilepsy is approximately 5-7 cases per 10,000 children from birth to 15 years of age [2] and despite the differences across studies, it is possible to rate the prevalence of epilepsy in children as 4-5/1,000. Epilepsy is a complex condition caused by a variety of pathological processes in the brain. It is characterized by occasionally (paroxysmal), excessive, and disorderly discharging of neurons that can be detected by clinical manifestations, EEG recording, or both.

The diagnosis of epilepsy is mainly clinical. The use of EEG is also requisite for the diagnosis and the classification of epilepsy. Pathophysiologically, there are many theories, based on animal models, about the generation of the seizures that implicate the excitation and inhibition of neuronal membranes and the role of some neurotransmitters (i.e. GABA). Generally the prognosis of epilepsy for remission is good but depends on the underlying cause. Antiepileptic drugs and surgery can control many types of epilepsy, but 20-30% of people with epilepsy have the benign genetic epilepsies that remit without treatment. Although most seizures in children are benign and result in no long-term consequences, increasing experimental animal data strongly suggest that frequent or prolonged seizures in the developing, immature brain result in long-lasting sequel [3].

Anti-epileptic drug treatments can result in significant power spectral differences of the epileptic patients when compared to a control group. Salinsky et. al. [4] and Tuunainen et. al. [5] have both analyzed spectral EEG changes in adult patients taking AEDs. Salinsky in particular has considered four occipital EEG measures including the peak frequency, median frequency and relative theta and delta power to analyze a group of patients with low seizure frequency who were either starting or stopping AED therapy. A set of cognitive tests and a structured EEG were performed before the change in AED consumption and 12-16 weeks after. When compared with a control group, the peak frequency captured differences in patients stopping or starting AEDs. For those stopping AEDs, the median frequency and the percentage theta power also gave significant differences. Similarly, Tuunainen et. al. captured differences in AED patients and control subjects. In this case they used the absolute and relative power as well as the peak power frequency at left occipital brain lobes as features extracted from a four second, eyes open, experimental setting. Results showed that the occipital peak alpha frequency was significantly lower in patients than in controls. Furthermore, the absolute power of the patient group was significantly higher at baseline in the control group, over all channels for the delta, theta, beta and total activity. Absolute alpha power was also found to be higher but this result was not significant.

Cognitive and behavioral changes in children with epilepsy are often encountered and these may be related to the epilepsy itself, the necessary use of antiepileptic drugs or a possible surgery, the probable brain dysfunction or damage associated with the seizures and social and family reasons [6]. Specifically, there is an association between attention-deficit/hyperactivity disorder (ADHD) and epilepsy revealed by many studies [7,8] but there are also other psychiatric disorders more commonly associated with epilepsy. Depression is considered to be the most frequent psychiatric disorder in patients with epilepsy and it is reported that children with epilepsy examined with the Child Depression Inventory showed elevated scores for depression [9]. Pellock estimated the prevalence of anxiety in children with epilepsy at 16% [10]. There also seems to be an association between autism and epilepsy in children, but a strong relation between epilepsy in childhood and aggressive or oppositional behavior has not been established [11]. Due to the potential long-lasting effects of epilepsy, it is important to detect and deal with symptoms as early as possible. To address this issue, we consider the diagnosis of children who experienced very few seizures in the past but who have no psychological findings or notable symptoms and whose EEG is visually diagnosed by a clinician as being normal. These children are highly probable to experience epilepsies in the future. Thus, the aim of this study is to develop reliable techniques for the extraction of biomarkers from EEG that indicate the presence of such controlled epileptic patterns. We compare two different approaches of localizing activity differences and retrieving relevant information to identify young children having controlled epilepsy from their non-epileptic counterparts. The first approach focuses on power spectrum analysis techniques using a signal representation approach such as Wavelets to elaborate on the differences in classification results. The second approach focuses on analyzing the functional coupling of cortical assemblies using the widely used magnitude squared coherence (MS-COH) measure and the bivariate autoregressive (AR) coherence (AR-COH) measure on the actual EEG signal

## Methods

### Subjects

The epileptic group under study consists of twenty children aged 9-13 (9 boys, 11 girls) children selected from the pool of Pediatric Neurology outpatient Clinics of two Hospitals in Heraklion-Crete-Greece, where they were diagnosed and followed at regular intervals. These children, referred to as controlled epileptic, were put under scrutiny because of their early symptoms but they had no clinical findings of brain damage or dysfunction and their EEG was visually normal. They had one or more epileptic seizures in the past and some of them were under monotherapy with drugs in low doses, without clinical side-effects. Inclusion criteria for patients and controls consisted of: a) age of 9-13 years old b) normal intellectual potential (assessed with WISC-III) c) absence of neurological damage-documented by neurological evaluation for patients and controls and by brain CT and/or MRI scan for patients and d) absence of psychiatric problems (based on parent's interview). These children were treated using common antiepileptic medication (in therapeutical doses without clinical side effects) only after they exhibited at least two seizures. The type of seizures diagnosed were the most common ones in childhood (Rolandic epilepsy, idiopathic generalized seizures, focal secondary generalized seizures without detectable brain damage and absence seizures). Written informed consent was obtained from the patients for publication of this case report and accompanying images. A copy of the written consent is available for review by the Editor-in-Chief of this journal.

### Recordings

**Figure 1:**
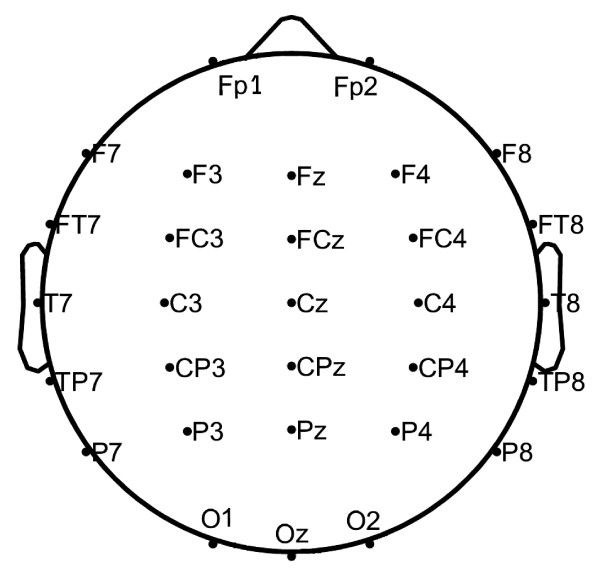
**Electrode montage consisting of 30 electrodes placed according to the 10/20 international electrode placement system**.

Continuous EEGs were recorded in an electrically shielded, sound and light attenuated room while participants sat in a reclined chair. The EEG signals were recorded from 30 electrodes placed according to the 10/20 international system, referred to linked A1+A2 electrodes. This electrode montage is shown in Figure 1. The signals were amplified using a set of Contact Precision Instrument amplifiers (Cambridge, MA, USA--http://www.psylab.com), filtered on-line with a band pass between 0.1 and 200 Hz, and digitized at 400 Hz. Off-line, the recorded data were carefully reviewed for technical and biogenic artefacts, so that only artefact free epochs of 10.24s duration are investigated. Artefacts were treated visually by an expert, since many automated artefact removal algorithmic methodologies, even if they are successful in removing certain types of artefacts, fail to leave physiological EEG intact. Thus, only pieces without visible artefacts (EOG, EMG, movements) were preserved. For each subject only one representative 10.24s epoch is included in the data. The selection of EEG epochs was performed blindly by an expert without knowing the group of each subject. Also the length of the epoch was chosen as it is short enough to assume stationarity and from the experience of our clinical lab, this period is enough for the analysis required [12,13]. The procedures used in the study had been previously approved by the University of Crete Institutional Review Board.

### Test description

In this study, two different tasks were analyzed. During the control (passive viewing) task (Task 1) subjects were at rest and had their eyes fixed on a on a small star displayed at the centre of a computer screen to reduce eye artefacts. The second task was a mathematical task (Task 2) involving the subtraction of two-digit numbers (e.g. 34 - 23, 49 - 32) [14], displayed on an LCD screen located in front of the participants at a distance of approximately 80 cm, subtending 2-4 degrees of horizontal and 2-3 degrees of vertical visual angle. Such a mental task is considered to be difficult for the studied age group. Vertical/horizontal eye movements and blinks were monitored through a bipolar montage from the supraorbital ridge and the lateral canthus. The analyzed epochs were acquired during the intensive calculation phase.

### Analysis

In this study two different approaches of localizing activity differences and retrieving relevant information for classifying the two children groups are compared. Section (4.1) focuses on power spectrum analysis techniques. In particular, we elaborate on the differences in classification results obtained when using Wavelets, which is a non-parametric approach that actually achieves an alternative signal representation [13]. Section (4.2) focuses on analyzing the functional coupling of cortical assemblies using the traditionally formulated but widely used *magnitude squared coherence *(MS-COH) and the coherence measure applied on a bivariate autoregressive (AR) process (AR-COH). Coherence is a normalized measure of linear dependence between two signals and is capable of identifying linear synchrony on certain frequency bands [15,12].

### Univariate power spectrum analysis

Features extracted from the time-frequency spectrum when using Wavelets are compared and their effect on the classification of the two groups is analyzed, while the subjects performed the control (rest) task (Task 1) and math task (Task2). Wavelets derive significant features encoding brain activity throughout the test period, which can also be localized in time for the study of abrupt or transient responses.

Biomarkers are constructed for specific brain regions (lobes) assuming a preselected lobe scheme that covers the entire head and is separated in groups of channels that are expected to function in a similar manner. The lobes (channel groups) considered are: FL (FP1, F3, F7), FR (FP2, F4, F8), CL (C3, CP3), CR (C4, CP4), PL (P3, P7), PR (P4, P8), TL (FT7, T3, TP7), TR (FT8, T4, TP8) and OL (O1, P7), OR (O2, P8). Furthermore six sequential frequency bands were considered in this analysis: delta (0-4 Hz), theta (4-8 Hz), alpha (8-13 Hz), beta (13-30 Hz), gamma1 (30-45 Hz) and gamma2 (45-90 Hz).

### Wavelet transform (WT)

The WT has developed into an important tool for analysis of time series that contain non-stationary power at many different frequencies (such as the EEG signal), and it has proved to be a powerful feature extraction method [16]. The epileptic recruitment rhythm during seizure development is well described in terms of relative wavelet energies [17]. The WT as compared to the FFT is more suitable for analyzing transient signals because both frequency (scales) and time information can be obtained in good resolution.

The continuous wavelet transform (CWT) was preferred in this work, so that the time and scale parameters can be considered as continuous variables. In the CWT the notion of scale *s *is introduced as an alternative to frequency, leading to the so-called time-scale representation. The CWT of a discrete sequence *x*_*n *_with time spacing *δt *and *N *data points (*n *= 0,1, ..., *N*-1) is defined as the convolution of *x*_*n *_with consecutive scaled and translated versions of the wavelet function *ψ*_0_(*η*):(1)(2)

where *s*, *η *and *ω*_0 _indicate scale, non-dimensional "time" and "frequency" parameters, respectively and . In our application, *ψ*_0_(*η*) describes the most commonly used wavelet type for spectral analyses, i.e., the normalized complex Morlet wavelet as given in (2). The frequency parameter *ω*_0 _is selected equal to 6 since it is a good trade-off between time and frequency localization for the Morlet wavelet. The wavelet function *ψ*_0 _is a normalized version of *ψ *that has unit energy at each scale, so that each scale is directly comparable to each other. There exists a concrete relationship between each scale *s *and an equivalent spectral frequency f, which for the Morlet wavelet is given by f = 1/(1.03 s) [18], so that scales can be mapped to frequency bands [13]. Thus, we can obtain the power spectrum of WT at specific frequency-scale *s *for each channel *c*, through the time-scale-averaged power spectrum . The corresponding biomarkers for each subject are obtained for each brain lobe *l *(which includes specific channels) and band *B *(which includes several scales), can then be computed as:(3)

where *c*_*l *_represents the set of channels within each lobe *l *and *s*_*B *_the number of frequency bins in band *B*. Notice that in the power measure we use the dB value.

### Bivariate synchronization analysis

In this study we also employ a methodology towards investigating the capabilities of linear measures in revealing the coupling between EEG channels in real band-limited signals. Synchronous oscillations of certain types of such assemblies in different frequency bands relate to different perceptual, motor or cognitive states and may be indicative of a wider range of cognitive functions or brain pathologies [19,20]. Hence, in the bivariate case we considered the MS-COH and the AR-COH measures and applied them in classifying the two subject groups, in the same analysis scheme as described in Section 3.1 for the univariate case. In this case a synchronization value is calculated between a selected pair of electrodes resulting in bivariate measures that can be treated similarly to the ones in the univariate case. Once the additional synchronization features are calculated they are fed to the classifier to discriminate between the two subject groups.

### Magnitude squared (MS-COH) and AR coherence (AR-COH)

For the time series *x*_*n *_and *y*_*n*_, *n *= *1 *... *N*, where *x*, *y *represent pairs of channels, the well-known expression of the Magnitude Squared Coherence (MS-COH) is given by:(4)

where *f *denotes frequency, *S*_*xy *_denotes the cross spectral density function, while *S*_*xx *_and *S*_*yy *_are the individual autospectral density functions for *x *and *y*, respectively [15]. To compute the MS-COH with nonparametric methods, we use the Welch's periodogram smoother, with a non-overlapping Hamming window of 1024 samples length. In the formula above, we employ the notation ⟨·⟩ to emphasize that window averaging is applied. Note that MS-COH for a given frequency *f *ranges between 0 (no coupling) and 1 (maximum linear interdependence). For each brain lobe *l *and band *B *the MS-COH (*γ*_*B, l*_) can be defined as the average of eq. 4, for *x*, *y *within the specific lobe and *f *within the specific band.

The linear dependence between the signals *x *and *y *can be modeled by a bivariate autoregressive (AR) process of order *m*. Let **Z**_*n *_= [*x*_*n *_*y*_*n*_]^T ^for 1 ≤ *n *≤ *N *and **z**_*n *_= [0 0]^T ^for *n *< 1, with the convention that ^T ^denotes transposition. Then we have **z**_*n *_= -**A**_1_**z**_*n*-1 _- ⋯- **A**_*m*_**z**_*n*-*m *_+ **e**_*n *_where the entries of the 2 × 2 matrices **A**_1_, ..., **A**_*m *_are real-valued. The residuals ***e***_*n *_are temporally uncorrelated and their covariance matrix is denoted **Q**_*m*_. The bivariate AR model leads to the following factorization of the spectral matrix [21]:(5)

where *i*^2 ^= -1, **A**_0 _is the identity matrix and the symbol * is used for conjugate transpose. For example, MS-COH can be readily computed, and we use the name AR-COH whenever the evaluation of the MS-COH is based on the spectral matrix factorization. A detailed description of algorithms for estimating **A**_1_, ..., **A**_*m *_and **Q**_*m*_, which are defined for specific *x*, *y *and *f *from EEG data, can be found [22]. The results reported in Section 4.2 have been obtained with the Whittle-Wiggins-Robinson estimation method [23,24]. The order of the autoregressions was selected from {1, ..., 50} by applying the Minimum Description Length criterion [25]:(6)

The band and lobe specific measure is defined similar to the corresponding MS-COH measure (i.e. *γ*_*B, l*_). The MS-COH and AR-COH synchronization values ranging from 0 to 1 are used as biomarkers in the bivariate case and are calculated for each brain region (lobe) assuming again a preselected lobe scheme that contain grouped channel pairs instead of single channels. The lobes (channel pair groups) for the bivariate case are: OPL (O1-P3, O1-P7, P7-P3), OPR (O2-P4, O2-P8, P8-P4), CPL (CP3-P3, C3-CP3, P3-P7), CPR (CP4-P4, C4-CP4, P4-P8) FTL (FP1-F7, FP1-F3, FT7-T3, FT7-TP7, T3-TP7), FTR (FP2-F8, FP2-F4, FT8-T4, FT8-TP8, T4-TP8), TL (FT7-T3, T3-TP7, FT7-TP7), TR (FT8-T4, T4-TP8, FT8-TP8).

### Feature Selection and Classification

This study proposes a statistical method for mining the most significant lobes using the available biomarkers, resembling the way many clinical neurophysiological studies evaluate the brain activation patterns. Since the goal is to find significant differences between two groups, the independent two-sample *t*-test is used to assess whether the means of the two groups are statistically different from each other. As a parametric test it assumes that: i) data comes from normally distributed populations, ii) data is measured at least at the interval level, iii) variances of the populations involved are homogenous and iv) all observations are mutually independent [26]. In this analysis, the feature vectors for control subjects (*F*_*C*_) and for epileptic subjects (*F*_*E*_) consist of the biomarkers *M*_*B, l *_which are the log-transformed values of the power (univariate case) or the synchronization values (bivariate case) within a specific frequency band *B *for a particular lobe *l*. Thus, the feature vectors are formed as:(7)(8)

where  or  represents the set of biomarker for control or epilepsy subject i (*Ci *or *Ei*), within frequency band *B*, and for a particular lobe *l*. In our application, the number of bands *B *ranges from one to six and the number of lobes *l *ranges from one to ten. These feature vectors can be defined for the various forms of biomarkers (wavelet power, MS-COH and AR-COH) defined above, or for combinations of measures. By using the D'Agostino Pearson test [26] or Kolmogorov-Smirnov's test [26], the features were found to have a normal distribution, thus satisfying assumption (i). Distance between points along the scale of the possible feature values was equal at all parts of the scale, thus ensuring that data is measured at least at the interval level (assumption (ii)). Homogeneity of variances was tested using Levene's test based on the *F*-statistic [26] and in this case it was found that the features from the two groups did not have equal variances. As this violates one of the above assumptions, the *t*-test had to be applied assuming unequal variances (Behrens-Fisher problem). Finally, since the biomarkers in *F*_*C *_and *F*_*E *_are coming from two independent groups (controls and epileptics) assumption (iv) is reasonable.

This statistical analysis technique was used to identify which lobes and frequency bands give significant differences between the epileptic subject group and the control group for both the signal representation approach and the signal modelling approach in the univariate and bivariate cases.

Once the features were available, classification was performed using a simple linear discriminant analysis (LDA) classifier with the leave-one-out validation approach. This means that one subject was tested while all the rest were used for training. In the results section we give the classification scores for the respective frequency bands and brain lobes to identify the number of correctly classified subjects out of the total population of 40 children. Apart from this the corresponding sensitivity and specificity measures are provided.

## Results

### Univariate power spectrum analysis

**Figure 2:**
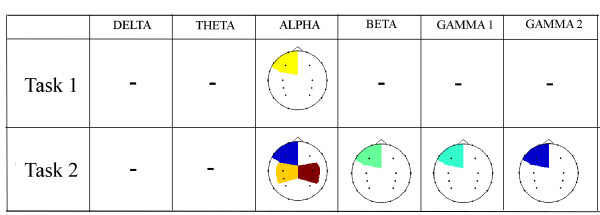
**Topographic maps showing the *p*-values of WT power differences between control and epilepsy subjects for Task 1 and Task 2**. The black dots in each image represent the channel locations. Lower *p*-values are indicated in shades of blue while *p*-values close to the threshold of 0.1 are indicated in shades of red. Blank areas within each topographic map indicate that the features extracted from that particular lobe do not give significant differences between the two populations (*p *> 0.1).

The WT was applied to the real EEG data, where each signal was initially set to zero mean and unit variance. In each case, we compute the lobe/band significance, as well as the corresponding classification scores with sensitivity-specificity measures. Figure 2 illustrates the topographic maps of the *p*-values between the two groups, obtained for each task and frequency band. Cells which have been left blank indicate no significant difference at the 90% confidence interval (*p *> 0.1). Shaded brain lobes represent a *p*-value ranging from 0.01 to 0.1, with shades of blue indicating the lowest *p*-values. These topographic maps show clearly that for the control task (Task 1) few brain areas have been identified by Wavelets to give significant differences between the two groups. The Wavelet approach detected significant differences in the left frontal lobe of the Alpha band only. Since frontal channels may easily be affected by eye movements, this result may be purely sporadic. Differences in the Alpha band are expected, since the Rolandic EEG rhythms at rest are dominated by Alpha and Beta activity [27].

However, for Task 2 the WT succeeds in identifying significant spectral differences within the frontal left lobes of Alpha and Gamma2 band and central lobes of the Alpha band. Alterations in the Alpha band are also expected since they are generally associated with problems in attention and episodic memory [28]. For higher frequency bands WT found low significant differences in left frontal areas. Differences at higher frequencies, particularly in the gamma bands, for such a cognitive task is probably related to the task complexity itself [29].

**Figure 3:**
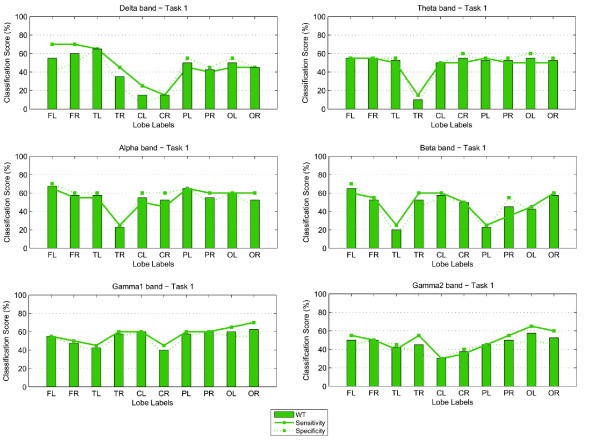
**Classification scores, Sensitivity and Specificity using WT features: Results for Task 1**.

**Figure 4:**
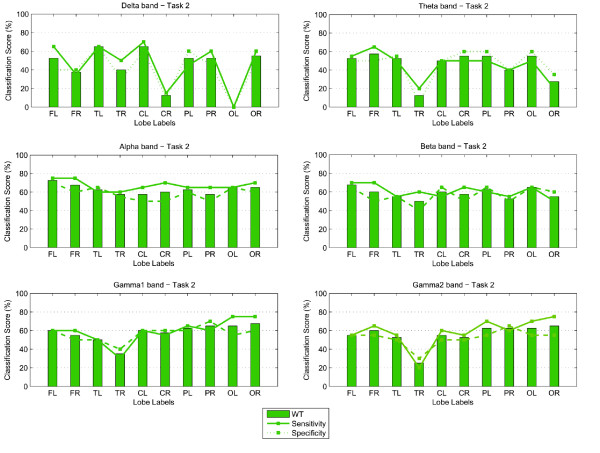
**Classification scores, Sensitivity and Specificity using WT features: Results for Task 2**.

The classification scores (percentage correct) and sensitivity-specificity measures for both Task 1 and 2, are shown in the form of bar graphs in Figures 3 and 4. A linear discriminant analysis (LDA) classifier with the leave-one-out evaluation scheme was implemented to derive the number of correctly classified subjects. In this case 39 out of a total of 40 children available were used for training while the remaining subject was then used in the testing process. The plots show that the spectral biomarkers for Task 1 result in classification scores close to 60%. The most consistent result across the different brain regions, for WT, occurred for the Theta and Alpha bands with the exception of the score over the right temporal brain region which fell well below chance level. The bar graphs also show that overall the Gamma1 band was consistent, as well.

For Task 2, the classification scores are more sporadic than those obtained for Task 1. The most stable result across the different brain lobes was obtained for the Alpha band (where the highest score of 72.5% was achieved) and the Beta band over the frontal lobe. For the Gamma bands, WT also obtained relatively stable scores over the parietal and occipital brain areas, but as shown in the topographic maps earlier, the occipito-parietal differences at these sites were not significant.

### Bivariate synchronization analysis

**Figure 5:**
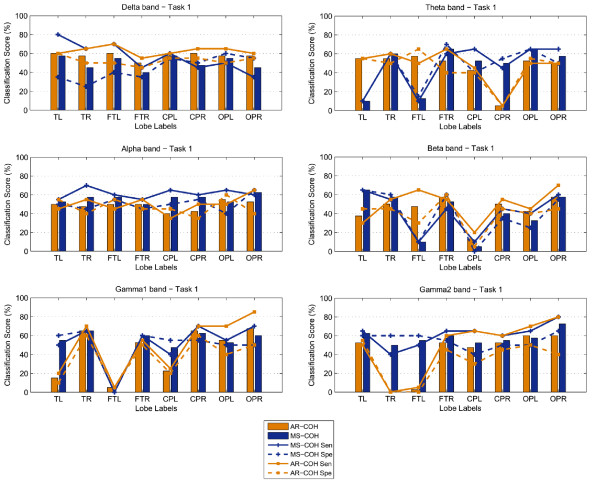
**Classification scores, Sensitivity and Specificity results using MS-COH and AR-COH features: Results for Task 1**.

**Figure 6:**
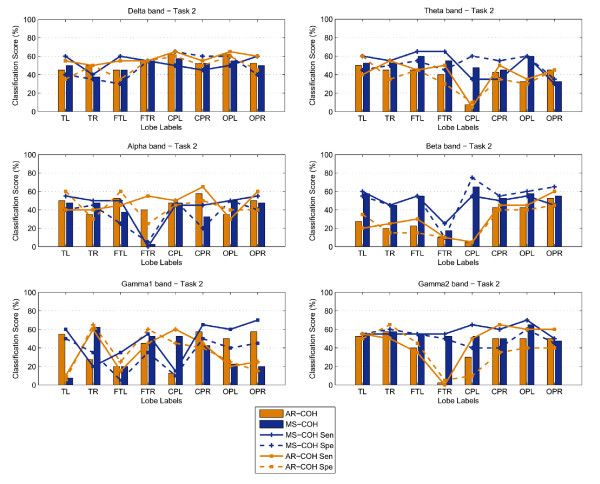
**Classification scores, Sensitivity and Specificity using MS-COH and AR-COH features: Results for Task 2**.

The MS-COH and AR-COH measures are computed on both "normal" and "controlled-epileptic" band-filtered data (using a fourth order zero-phase shift bandpass Butterworth filter). Similar to the results of the previous section, the classification scores and sensitivity-specificity measures for MS-COH and AR-COH, for Tasks 1 and 2 are shown in the form of bar graphs in Figures 5 and 6, respectively. The plots show that the maximum classification score achieved for Task 1 was in the Gamma2 band for the occipito-parietal lobes (OPL, OPR), where 72.5% classification was reached (MS-COH). For Task 2, the maximum classification score achieved was 65% (MS-COH) in CPL - Beta band and OPL - Gamma2 band. Even if this score is low, a general trend observed in Figures 5 and 6 is that the central-parietal (CPL-CPR) and occipito-parietal (OPL-OPR) lobes achieve overall better scores. As a final step towards a better classification result for Task 2, we considered fusing selected biomarkers from the univariate and the bivariate case (see section 3.3.3). Finally, it should be noted that nonlinear measures (phase and generalized synchronization) were also tested but not included in this paper since they were not able to identify any statistically significant differences.

### Selection of biomarkers

#### Biomarkers based on WT

**Figure 7:**
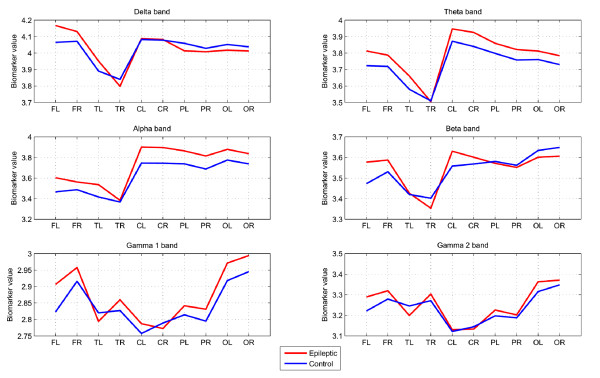
**Averaged WT biomarkers across the 20 epileptic and 20 control subjects, for each frequency band and brain lobe considered**.

As discussed previously, WT derives good classification estimates for feature selection in Task 1. This task operates similar to [19] in an "eyes open" scheme. Attempting a comparison with this previous work, in Figure 7 we illustrate the WT biomarkers averaged over the 20 epileptic and 20 control children respectively across different frequency bands and brain regions.

For controlled epileptic children our analysis derives consistent higher energy in the Theta and Alpha bands, as well as a symmetrical energy variation pattern in Delta, Theta, Alpha and Beta bands. This result is in line with earlier studies [19,30], which found an increase in delta-theta ranges (3-7 Hz) and upper Alpha-lower Beta ranges (15-17 Hz) in patients with partial and generalized epilepsies. From this relation and the significant areas derived by WT analysis, we select Theta-Alpha band activity on central and temporal channels (TL, TR, CL and CR) for further analysis of our results from univariate analysis. In Table 1 we analyze the spectral biomarkers of the two groups for Task 1. Specifically, the table presents the average and the standard deviation values of the biomarkers across the analyzed brain lobes, for the epileptics and controls, respectively. Results for each of the six frequency bands are tabulated. These results verify that on average, the epileptic children had significantly higher spectral biomarkers, especially on the Theta and Alpha bands where the difference is shown to be the most significant (p < 0.5).

**Table 1:** Average WT Biomarker Values of lobes (TL, TR, CL, CR) for Task 1.

	Epileptics	Controls	*p*-values
**Delta**	3.98 ± 0.24	3.97 ± 0.31	0.94

**Theta**	3.76 ± 0.28	3.70 ± 0.20	0.45

**Alpha**	3.68 ± 0.29	3.57 ± 0.24	0.20

**Beta**	3.50 ± 0.19	3.49 ± 0.19	0.80

**Gamma1**	2.80 ± 0.22	2.80 ± 0.21	0.94

**Gamma2**	3.19 ± 0.17	3.20 ± 0.21	0.94

The largest difference occurred within the Alpha band, as was expected for a child group where the spectral peak may also spread into the theta band, since in children different frequency bands are not yet functionally differentiated and separated from the broad alpha frequency range and, thus responds more in an alpha-like way [31]. Relative to an age matched control group, epileptic patients between 9 and 11 years analyzed in [32] have also shown an increase in theta and alpha power.

When considering the mathematical subtraction Task 2 the most significant bands (Table 2) are Theta, Beta and Gamma2. In comparison with the rest Task 1 in each group, we would expect to find increased power activity in Gamma as well as Alpha frequency bands. There is extensive evidence that neural oscillations increasing power in the Gamma band are involved in the visual perception of objects and correlate with cognitive task assignments [29,33]. Furthermore, children with epilepsy have been reported to reflect alterations in the Theta band in tasks associated with attention and episodic memory [28]. Considering the derived classification estimates for Task 2, we also find evidence of differences in these bands through the WT analysis. In Section 3.3.3 we further consider fusion of biomarkers in an attempt to increase the overall discrimination ability.

**Table 2:** Average MS-COH Biomarker Values of lobes (CPL, CPR, OPL, OPR) for Task 2.

	Epileptics	Controls	*p*-values
**Delta**	0.76 ± 0.10	0.74 ± 0.11	0.57

**Theta**	0.71 ± 0.12	0.68 ± 0.11	0.44

**Alpha**	0.69 ± 0.12	0.69 ± 0.09	0.89

**Beta**	0.69 ± 0.10	0.66 ± 0.13	0.34

**Gamma1**	0.72 ± 0.15	0.72 ± 0.10	0.97

**Gamma2**	0.73 ± 0.11	0.77 ± 0.07	0.16

#### Biomarkers based on synchronization measures

For both tasks the synchronization measures lead to slightly inferior classification estimates compared with the univariate (power) measure. Thus, the selection of synchronization measures for further consideration has been associated with that of power measures and also directed by the existing literature. In general MS-COH appears more efficient than AR-COH in exemplifying small differences. Task 1 does not indicate any significant difference between the two studied groups, based on MS-COH. In association with the selection of WT features in Section 3.3.1, we further consider synchronization measures in the Theta and Alpha bands (Table 1, *p *< 0.5), with the aim of exploring the fusion of both power and synchronization biomarkers in enhancing classification scores (Section 3.3.3).

For the cognitive (subtraction) process in Task 2, we would expect some increased synchronization especially in the gamma band, where synchronous localized and/or broadband rhythmic bursting in assemblies of neurons are associated with several consciousness processes [29] and present increased activity in people with partial or generalized epilepsy [19]. In our analysis (Figure 6), differences in classification scores based on synchronization are low and insignificant. Further analysis based on average measures per group has been performed on lobes expressing the highest classification scores. More specifically, Table 2 summarizes the average biomarkers for both the epileptic and control groups in all six frequency bands for a lobe subset consisting of CPL, CPR, OPL and OPR. The results show that the biomarker values for the two groups are close to each other and hence not significant. From Table 2, the highest *p*-value for discrimination is achieved in the higher Gamma band, followed by the Beta band. The latter also gives better overall classification scores in Figure 6. There is further evidence of the involvement of the Beta band in cognitive tasks in a way similar to that of Gamma band, however with weaker enhancement of activity [29]. Thus, even though on its own MS-COH fails to distinguish between the epileptic and control children, we further consider the Beta band at lobes CPL, CPR, OPL and OPR for further consideration in a fusion strategy along with power measures, as described in the next section.

### Decision support for controlled epilepsy based on EEG biomarkers

In order to summarize the above results in the decision framework and use potential biomarkers in such a way as to increase differentiation between the two groups, we consider a fusion scheme for the available features. Task 1 and Task 2 were considered separately, in order to involve the most prominent features as biomarkers in each case.

Fusion tests were performed on three sets of features: power (WT) features only, MS-COH features only and a combination of power and MS-COH features. Four simple fusion operators were tested as follows:

1 A Linear Discriminant Classifier (LDC) applied to the average of all selected features

2 A majority vote function applied on the classification outcomes of selected biomarkers. This decision function selects the class label based on which of the available classes (epileptic or normal) gets more than half the votes.

3 A weighted sum of individual classification scores.

4 The MINDIST Algorithm which calculates the least squares distance to the average of features inside each known class, i.e. epileptic or normal and assigns a label based on that class with the minimum distance.

For Task 1, the individual classification scores obtained from specific lobes and frequency bands are not satisfactory. Across all lobes, the best results obtained are those for WT, with an average classification score of 54.5%, 54.3% and 49% across the Alpha, Gamma1 and Theta bands respectively. Over the same bands MS-COH obtained classification scores of 46.6% and 56%. Based on these results and in an attempt to also relate with the widespread distribution of differences derived in [19] between epileptics and controls during the eyes-open, rest state, we included in a fusion scheme features from WT and MS-COH analysis related to the theta and alpha bands. When considering a total of 10 features, 7 from the WT approach (FL, FR, CR, OL from the theta band and FL, PL, OL from the alpha band) and 3 features from the MS-COH approach (FTR, OPL from the Theta band and OPR from the Alpha band), the best classification score reached 65% with a sensitivity and specificity measure of 70% and 60% respectively (Table 3). The choice of features was based on the criteria of highest individuals and specificity/sensitivity measures higher than 50%. Although this fusion result shows a slight improvement over individual features, the classification is still reasonably low. A further rigorous feature selection process resulted in five specific features to be fused, two WT features (FL and PL from the Alpha band) and three MS-COH (FTR, OPL from the Theta band and OPR from the Alpha band. These gave a score of 80% (Table 3) which is now superior to the 65% obtained earlier. This result shows that fusion of features in the Theta-Alpha bands can yield significant improvements in classification scores over individual scores. Hence, this is our proposed strategy for designing a decision support system that can efficiently detect particular characteristics of children with epilepsy.

**Table 3:** Task 1: Best Results of fusion based on selected features from WT + MS-COH.

	Fusion operator	Sensitivity	Specificity	Classification score
WT+ MS-COH (# of features: 10)	LDC on Average	70%	60%	65%

WT + MS-COH (# of features: 5)	Majority Vote	80%	80%	80%

WT + MS-COH (# of features: 29 All WT with scores ≥57.5 + all with scores ≥ 57.5 from MS-COH)	Majority Vote	60%	70%	65%

WT + MS-COH symmetric combination choice based on High classification score (non algorithmic choice) WT: FL, FR, PL, PR MS-COH: OPL, OPR	LDC on Average	80%	50%	65%

For Task 2, the individual classification scores obtained from specific lobes and frequency bands are even lower. Across all lobes and frequency bands, the best results obtained are those for Wavelets, with an average classification score of 54% and those for MS-COH with an average classification score of 47%. We further explored the potential of fusing biomarkers in order to increase the discrimination ability. A total of 20 features were selected, 16 features from the WT approach (alpha: PL, OL, OR; beta: CL, PL, OL; gamma1: CL, CR, PL, PR, OL, OR; gamma2: PL, PR, OL, OR) and 4 features from the MS-COH approach (beta: CPL, CPR, OPL, OPR). The selection of these features was based on the best classification scores and sensitivity or specificity measure higher than 50%. The results obtained when applying these operators to the various feature sets for Task 2 are shown in Table 4.

**Table 4:** Task 2: Best Results of fusion based on selected features from WT, MS-COH and WT + MS-COH.

	Fusion operator	Sensitivity	Specificity	Classification score
WT (# of features: 16)	LDC on Average	80%	60%	70%
	
	Majority Vote	65%	65%	65%
	
	Weighted Sum	70%	60%	65%
	
	MINDIST	80%	60%	70%

MS-COH (# of features: 4)	LDC on Average	60%	50%	55%
	
	Majority Vote	55%	75%	65%

WT + MS-COH (# of features: 20)	Mindist (WT) or MajorityVote (MS-COH)	80%	60%	70%

The highest classification scores for the WT features reached 70% which is still lower than the 72.5% score obtained by WT over lobe FL for the alpha band. The results for the MS-COH features yield even lower scores. Finally, when univariate and bivariate features were combined a score of 70% was once again achieved. Overall, fusion of features did not result in any significant improvement in classification scores over the individual scores achieved for Task 2. Thus no biomarker was found to reliably discriminate between the two groups while the subjects are performing this mathematical task. Nevertheless, a related work reported during the review process of this paper reveals that other auditory tasks related to episodic memory have shown potential in classifying a group of children with mild signs of epilepsy [28]. Thus, a more rigorous consideration of various tasks should be performed towards the design of a decision support system, which can reflect wider aspects on the performance of children with epilepsy.

## Discussion and Conclusion

This work considers methods for the discrimination of a controlled epileptic child group and an age-matched control group. The children considered in this analysis are at an age range where maturing is not drastic and education is not significantly different. Thus, we expect only small differences due to age. The experiment is in a matched controls scheme where we have same numbers in the two groups in terms of age, sex and education. The studied population of controlled epileptic children does not show clinical dysfunction or other EEG abnormalities. We are using sensitive methods of analysis in order to search for signs of differences from age-matched controls. Such signs are indicative of slight neurophysiological disturbances that are not obvious in usual neuropsychological tests and electrophysiological EEG recordings. Even though these disturbances are not considered serious, the children need to follow a certain course of therapy and follow-up in order to restrict their effects.

Our first aim was to check whether controlled-epileptic children exhibit spectral differences in their EEGs in comparison to an age-matched control group during a control situation and while performing a mental task. Secondly, we address the development of sensitive and reliable measures for discrimination between the two groups by means of either power spectrum *univariate *measures or *bivariate *synchronization measures of different brain regions or both. The latter stems from the fact that neuronal dynamics and synchronization phenomena have been increasingly recognized to be important mechanisms by which specialized cortical and sub-cortical regions integrate their activity to form distributed neuronal assemblies that function in a cooperative manner [34]. According to our knowledge, such an integrated analysis has not been carried out so far.

Clinical and psychological examinations, as well as visual EEG inspection, do not provide any information leading to differences. On the original EEG data we apply two types of methodologies, one based on the power spectrum using direct signal representation (through wavelets), and the other on capturing the coupling of different lobes using linear synchronization indexes (MS-COH and AR-COH). The extracted features in each lobe and band are examined through significance tests, classification accuracies and statistical distributions of biomarkers.

The results of this paper indicate that univariate Wavelet analysis, as well as bivariate synchronization analysis based on MS-COH, can provide different features for discrimination. Thus, such methods could be used in a complementary manner towards the design of a decision support system aimed at detailed neurophysiological assessment. Fusion of selected biomarkers in the Alpha bands resulted in an increase of the classification score up to 80% (Table 3) during the rest condition. No better discrimination (70%-Table 4) was achieved during the performance of a cognitive subtraction task. Other recent studies have illustrated discrimination during tests triggering episodic memory. These results, however, need further investigation, particularly on a larger dataset and follow-up of many years, to be able to state concretely which brain areas and frequency bands can best assess slight brain dysfunction in cases of controlled epilepsy and perhaps in other disturbances of neurophysiological origin.

## Competing interests

The authors declare that they have no competing interests.

## Authors' contributions

VS, TC and MZ were responsible for the design of the study and writing down the manuscript. VS, TC and MZ, CDG conducted the univariate and bivariate analysis, respectively. VS, TC and CB conducted the feature selection, classification and classifier fusion processes, respectively. SM and EK conducted data acquisition and interpretation. KPC and SGF helped to draft the manuscript. TC and KM worked on the charts and illustrations creation. All authors read and approved the final manuscript.
